# Carotid artery atherosclerosis, low and high volumes of high-intensity interval training in patients after myocardial infarction: the precision of measurement embarks on a precise measurement protocol

**DOI:** 10.1177/17539447251318932

**Published:** 2025-02-08

**Authors:** Christian Saleh

**Affiliations:** University of Basel, Petersplatz 1, Basel 4031, Switzerland

**Keywords:** atherosclerosis, carotid artery, carotid intima-media thickness, high-intensity interval training, surrogate marker, ultrasound

Aispuru-Lanche et al. published in a recent issue a study titled, “Vascular-endothelial adaptations following low and high volumes of high-intensity interval training in patients after myocardial infarction.”^1^ The authors wrote that endothelial dysfunction and oxidative stress could be attenuated by high-intensity aerobic interval exercise training (HIIT).^1^ The study aimed to evaluate “the impact of two volumes of HIIT, low (LV-HIIT, <10 min at a high intensity) and high (HV-HIIT, >10 min at a high intensity), on vascular-endothelial function in individuals after an acute myocardial infarction (AMI).”^1^ Included were 80 patients with AMI (58.4 ± 8.3 years, 82.5% men) “with three study groups: LV-HIIT (*n* = 28) and HV-HIIT (*n* = 28) with two sessions per week for 16 weeks and control group (CG, *n* = 24) with unsupervised physical activity recommendations.”^1^ As a surrogate marker for preclinical atherosclerosis, the carotid intima-media thickness (cIMT) was determined before and after the intervention period.^1^ The authors stated, “The assessment of cIMT enabled us to gauge the extent of atherosclerosis among the study participants. The average cIMT within the sample stood at 0.675 ± 0.183 mm, and there were no discernible differences at baseline among groups. Following a 16-week HIIT intervention, there were noteworthy yet slight alterations in carotid atherosclerosis means compared to the attention control (AC) group (ANOVA *p* = 0.019). Participants in the LV-HIIT program experienced a mean reduction of −3.0 ± 1.1% (*p* = 0.0389), while those in the HV-HIIT program exhibited a mean reduction of −3.2 ± 1.2% (*p* = 0.0305).”^1^ Two comments (within the predefined brevity of a letter) are fundamentally needed to evaluate the cIMT results of this study.^1^ The authors wrote, “*To maintain consistency, parameters such as depth of field, gain, input power, dynamic range, monitor intensity, and other instrumentation settings were documented for replication in subsequent visits*.”^1^ However, the most important documentation for the follow-up ultrasound cIMT measurements was missed by the authors, namely marking the segment where precisely the baseline cIMT measurement occurred. “*The measurements were conducted on the posterior wall of both common carotid arteries, positioned between 1 and 2 cm from the carotid bifurcation, . . .*,” this, by authors^1^ described method, is a highly imprecise and unreliable “marker” to delineate the measurement areal and unavoidably will lead to selecting a different areal (*also within the 2 cm margin!*) for the follow-up that may present with other wall characteristics. Important in the evaluation of atherosclerosis is that *atherosclerosis while a systemic disease presents in an asymmetric fashion*.^2^ It is important to recall, when we are using cIMT as a surrogate marker for preclinical atherosclerosis that cIMT values are expressed at the *sub-millimetric range* (the cut-off provided by the authors was 0.8 mm),^1^ consequently, smallest differences in cIMT values are equally sufficient to categorize subjects into different cIMT categories, from which important clinical, therapeutic or pathophysiological conclusions are deduced. The by the authors^1^ observed cIMT differences between follow-up and baseline examination can be the result of having selected a slightly different carotid artery areal for the follow-up measurement with other wall characteristics (which potentially were *already present at baseline!*): consequently the observed cIMT values in the follow-up examination can be totally *independent* of the performed intervention (here, HIIT). As the authors^1^ apparently also did not synchronize with the cardiac cycle (i.e., end-diastolic phase),^3^ they introduced a further major measurement bias, as cIMT measures unavoidably were made *randomly* in both cardiac phases, rendering the measures cross-sectionally and longitudinally for the same subject and between the subjects, incomparable. Mean cIMT differences between systole (vessel diameter expansion) and diastole (vessel diameter reduction) of 0.037–0.041 mm were reported.^4,5^ Considering these multiple and critical flaws in cIMT evaluation, the cIMT data and related conclusions of the Airspuru-Lanche et al. study^1^ should be considered with caution.

## Declarations

**Ethics approval and consent to participate:** Not applicable.

**Consent for publication:** Not applicable.

**Availability of data and materials:** Not applicable.
